# Does counselling improve uptake of long-term and permanent contraceptive methods in a high HIV-prevalence setting?

**DOI:** 10.4102/phcfm.v7i1.779

**Published:** 2015-11-06

**Authors:** Amon Siveregi, Lilian Dudley, Courage Makumucha, Phatisizwe Dlamini, Sihle Moyo, Sibongiseni Bhembe

**Affiliations:** 1 Mankayane Government Hospital, Swaziland; 2 Division of Community Health, Department of Interdisciplinary Health Sciences, Stellenbosch University, South Africa; 3 Institute of Development Management, Mbabane, Swaziland; 4 Hlatikhulu Government Hospital, Swaziland; 5 Piggs Peak Government Hospital, Swaziland

## Abstract

**Background:**

Studies have shown a reduced uptake of contraceptive methods in HIV-positive women of childbearing age, mainly because of unmet needs that may be a result of poor promotion of available methods of contraception, especially long-term and permanent methods (LTPM).

**Aim:**

To compare the uptake of contraceptive methods, and particularly LTPM, by HIV-positive and HIV-negative post-partum mothers, and to assess the effects of counselling on contraceptive choices.

**Setting:**

Three government district hospitals in Swaziland.

**Methods:**

Interviews were conducted using a structured questionnaire, before and after counselling HIV-negative and HIV-positive post-partum women in LTPM use, unintended pregnancy rates, future fertility and reasons for contraceptive choices.

**Results:**

A total of 711 women, of whom half were HIV-positive, participated in the study. Most (72.3% HIV-negative and 84% HIV-positive) were on modern methods of contraception, with the majority using 2-monthly and 3-monthly injectables. Intended use of any contraceptive increased to 99% after counselling. LTPM use was 7.0% in HIV-negative mothers and 15.3% in HIV-positive mothers before counselling, compared with 41.3% and 42.4% in HIV-negative and HIV-positive mothers, respectively, after counselling. Pregnancy intentions and counselling on future fertility were significantly associated with current use of contraception, whilst current LTPM use and level of education were significantly associated with LTPM post-counselling.

**Conclusion:**

Counselling on all methods including LTPM reduced unmet needs in contraception in HIV-positive and HIV-negative mothers and could improve contraceptive uptake and reduce unintended pregnancies. Health workers do not always remember to include LTPM when they counsel clients, which could result in a low uptake of these methods. Further experimental studies should be conducted to validate these results.

## Abstrait

**Les services de conseils contribuent-ils à améliorer l’utilisation permanente et à long terme des méthodes de contraception dans les zones à forte prévalence de VIH?**

**Contexte:**

Les études ont montré une diminution de l’utilisation des méthodes contraceptives chez les femmes séropositives en âge d’avoir des enfants, surtout en raison des besoins non satisfaits résultant de la mauvaise promotion des méthodes de contraception disponibles, notamment les méthodes permanentes et à long terme (MPLT).

**Objectif:**

Comparer l’utilisation des méthodes contraceptives, notamment les MPLT, par les mères séropositives et séronégatives venant d’accoucher, et évaluer les effets des conseils donnés sur le choix de contraceptif.

**Lieu:**

Trois hôpitaux de district gouvernementaux au Swaziland.

**Méthodes:**

On a mené des entrevues à l’aide d’un questionnaire structuré, avant et après avoir conseillé les femmes séropositives et séronégatives venant d’accoucher dans l’utilisation des MPLT, sur les taux de grossesses non voulues, la fécondité ultérieure et les raisons du choix de contraception.

**Résultats:**

711 femmes, dont la moitié était séropositives, ont participé à l’étude. La plupart d’entre elles (72.3% séronégatives et 84% séropositives) utilisaient des méthodes modernes de contraception, avec la majorité utilisant des médicaments injectables to les 2 ou 3 mois. Après consultations, l’usage prévu des contraceptifs a augmenté à 99%. L’utilisation des MPLT était de 7.0% chez les mères séronégatives et de 15.3% chez les mères séropositives avant la séance de conseil, comparé à 41.3% de mères séronégatives et 42.4% de mères séropositives, respectivement après la séance de conseils. Les intentions de grossesse et les conseils sur la fécondité ultérieure étaient liés considérablement à l’utilisation actuelle de contraception, alors que l’utilisation actuelle des MPLT et le niveau d’éducation étaient liés considérablement aux MPLT après la consultation.

**Conclusion:**

Les conseils donnés sur toutes les méthodes, y compris les MPLT ont réduit les besoins de contraception non satisfaits chez les mères séropositives et séronégatives t pourrait améliorer la prise de contraceptifs et réduire les grossesses non volontaires. Le personnel de santé ne se souvient pas toujours de parler des MPLT quand ils conseillent leurs clients, ce qui pourrait causer la faible utilisation de ces méthodes. Il faudra faire d’autres études expérimentales pour valider ces résultats.

## Background

Strategies for controlling the HIV and AIDS pandemic include reduction of the spread occurring through sexual intercourse, prevention of mother-to-child transmission (PMTCT), treatment of sexually transmitted infections, reduction in gender-based violence and prevention of unwanted pregnancies.^1,2^ An unintended pregnancy is a pregnancy that is mistimed, unplanned or unwanted at the time of conception.^2^ Globally, up to 50% of pregnancies are unintended, and as many as one-third of the 357 000 maternal deaths have been attributed to unintended pregnancies; despite its impact on maternal and child health, the contraceptive aspect of HIV prevention has been neglected.^3^ Preventing unintended pregnancies by contraception reduces perinatal transmission of HIV and is a cost-effective component of PMTCT.^2^ A study in eight African countries showed that a moderate decrease in the number of pregnancies in HIV-positive women resulted in the same number of HIV-infected births averted, as a result of the current PMTCT efforts by using nevirapine.^4^

Before the advent of antiretrovirals (ARVs), HIV-positive women had a short and poor quality of life, and most would not consider having children because of poor health or would not be fertile because of the disease.^5^ With ARVs, their quality and duration of life have improved.^5^ Fertility has also been shown to improve as immunological function improves.^6^ Many HIV-positive women choose, or are pressurised by family members, to have children.^7^ HIV-positive women of childbearing age are, however, not adequately counselled on sexuality and fertility intentions,^2,8^ resulting in unintended pregnancies and leading to unsafe abortions or deterioration of health, especially when the mother's viral load is high. It is therefore important that healthcare workers counsel HIV-positive women on future fertility plans and suitable contraception methods to avoid unintended pregnancies.

Underuse and inconsistent use of contraceptives contribute to unintended pregnancies.^9^ Evidence is lacking on the best method of contraception for HIV-positive women, but most authorities advocate long-term and permanent methods (LTPM) of contraception such as the intrauterine device (IUD), implants and sterilisation.^5^ A study in England showed a decrease in unintended pregnancies between 1998 and 2011, and a statistically significant association between the decrease and the use of LTPM was found.^10^ In 2012, the American College of Obstetricians and Gynaecologists recommended the use of LTPM for all women.^11^ These methods are safe, non-user-dependent and have the highest continuation rates compared with other methods.^12^ With LTPM, fertility can be delayed to a time when the couple feels ready, or when the woman's immune system has improved, without the risk of forgetting to take a pill or without having to come for 2-monthly injections.^8^ LTPM also has the advantage of reducing the pill burden as some of the HIV-positive women will be taking additional medication for comorbidities.

LTPMs are underutilised because of poor promotion of these methods by healthcare workers, with most women, especially those living with HIV and AIDS, using injectables and pills.^13^ Studies have, however, shown that proper counselling by healthcare providers improves acceptability of these methods.^9,14,15^ A study in Rwanda and Zambia showed an increased uptake of LTPM of up to 36% after contraceptive and fertility counselling in post-partum mothers.^16^ A randomised control study (RCT) was conducted in North Carolina, where post-partum mothers were randomised to receiving a script with LTPM information whilst the other group was not given anything. At follow-up, no significant difference was noted in the uptake of LTPM between the two groups.^17^ A similar RCT was conducted in Chicago, where participants were randomised to watching a video with LTPM information or a placebo video. The uptake of LTPM was also not significantly different between the two groups.^18^ We therefore chose to do face-to-face interviews with participants, giving them information on LTPMs and all the other contraceptive methods.

Swaziland has an HIV-prevalence of 26% in adults, with 31% in women of reproductive age and 42% in pregnant women.^19^ Women account for approximately 60% of cases, and there is a high rate of discordant couples.^20^ Approximately 50% of Swazi women of childbearing age are on contraceptives, with only 1.3% of these on IUDs, and 5.7% have been sterilised.^20^ Studies have shown a higher percentage of contraceptive uptake in women who have disclosed their statuses to spouses than those who have not.^21^ Disclosure is difficult for many women in Swaziland who are not in stable relationships.^19,22^

Female sterilisation is actively promoted in HIV-positive women in Swaziland government hospitals to an extent that some women feel coerced and regret having been sterilised.^7^ Promotion of IUDs and implants is neglected in both HIV-positive and -negative women.^15^

The present study therefore sought to describe the current use of contraceptive methods, particularly LTPMs, amongst post-partum HIV-positive and HIV-negative women in Swaziland, and to assess whether counselling improved the preference for future use of LTPMs.

## Methods

### Study design and setting

This was a before-and-after observational-analytic study of contraception uptake and factors associated with contraceptive uptake (mainly LTPMs) amongst HIV-positive and HIV-negative post-partum mothers going for treatment to three district hospitals in Swaziland. The three hospitals, in Mankayane, Hlatikhulu and Piggs Peak, have a burden of HIV of up to 42% in pregnant mothers.^11^ These are the only government district hospitals in the country that attend to similar patients in terms of numbers and characteristics. Pills, injectables, condoms, implants, IUDs and sterilisation are available at the three hospitals, with all contraceptives being given by nurses, except for sterilisation, which is performed in theatre by medical officers.

Contraceptive and LTPM preferences were measured before and after a 20-minute face-to-face counselling session on all available contraceptives by trained counsellors. The counsellors, who were also nurses from different departments of the same hospital, were trained for a week at Mankayane Government Hospital on all available contraceptives, advantages and disadvantages of each method, and indications and contraindications. They were also trained on standardised counselling techniques. A post-test was given after the course, which they all passed.

### Sample and study population

The study population included all women going to the public health units of the three hospitals for post-partum and child immunisation services up to 3 months post-partum. The trained data collectors screened the mothers coming to the facilities and included all who met the inclusion criteria and consented to participate. Mothers who had undergone hysterectomy were excluded.

### Inclusion criteria

Women coming to the hospitals’ public health units for a postnatal review and immunisation of babies up to 3 months were included in the study.

### Exclusion criteria

Women who did not test their HIV status in the previous pregnancy and those who underwent hysterectomy were excluded from the study.

### Sampling technique

A convenience sample of all consecutive women going to the hospitals for their 7 days’ and 6 weeks’ postnatal review and immunisation of their children up to 3 months were recruited and counselled until the required sample size was attained.

The attending women included those who had used PMTCT facilities in the previous pregnancy (the pregnancy for which they were attending postnatal services) and were presumed to be HIV-positive. PMTCT facilities have a testing rate above 99% for pregnant women and provide ARVs during pregnancy and labour and nevirapine to the babies. Inclusion criteria were the same for HIV-positive and HIV-negative mothers.

A sample size of 690 participants was calculated to detect a 2.5% difference in the use of LTPM between HIV-positive and HIV-negative mothers and a 10% difference in the uptake of LTPM before and after counselling. Previous studies have estimated the prevalence of LTPM to be around 7% in HIV-positive women and 5% in HIV-negative women.^6^

### Data collection

A staff nurse from each hospital was trained to conduct interviews with participants, in the patient's home language using a structured questionnaire. Study participants were interviewed by a separate trained data collector in each site before and after the counselling session. Data collected included demographics, time since last pregnancy, unintended pregnancy, previous exposure to contraceptive and LTPM counselling, and regret about using LTPM. Data were entered into Microsoft Excel by a data entry technician and checked by the principal investigator. Data were collected between February and May 2014.

### Data analysis

Data analysis was carried out using STATA 12 software. Medians, interquartile ranges and frequencies were used to describe the data. Fisher's exact test was used to compare the baseline characteristics between HIV-positive and HIV-negative women. Differences in contraceptive use between HIV-positive and HIV-negative women were compared by using Fisher's exact test. LTPM use before, and LTPM preference after, counselling were compared between HIV-positive and HIV-negative participants using chi-squared tests. Univariate and logistic regression analyses were performed to analyse factors associated with LTPM use before, and LTPM preference after, counselling. Univariate analysis was carried out using factors associated with the use of LTPM in the literature. Factors found to be associated with the outcome were included in the regression model. Missing values were excluded from the analysis.

### Ethics

The research was approved by the Stellenbosch University Ethics Committee (Reference number: S13/07/131) and Ministry of Health Swaziland Ethics Committee (Reference number: MH/599c/FWA00015267/IRB00009688).

## Results

A total of 711 women, of whom 359 were HIV-negative and 352 HIV-positive, participated in the study. The study participants were between 13 and 55 years old, with HIV-positive mothers being significantly older than the HIV-negative mothers (Table 1). More HIV-positive women were either formally employed or self-employed, knew their partners’ status and had more children than the HIV-negative women. Time since last pregnancy was the same between the two groups (Table 1).

**TABLE 1:** Summary characteristics of HIV-positive and HIV-negative mothers seeking post-partum services.

Characteristic	Response	HIV-positive *n* (%)	HIV-negative *n* (%)	Total *n*	*p*-value
Participants	-	352	359	711	-
Age in years Median 26 (interquartile range 21–31)	< 25	113 (35.0)	210 (65.0)	323	< 0.0001
	25–40	222 (61.)	142 (39.0)	364	
	> 40	17 (70.8)	7 (29.2)	24	
Employment status	Employed/sed/u	128 (54.2)	108 (45.8)	236	0.003
	Self-employed	60 (60.0)	40 (40.0)	100	
	Unemployed	164 (43.7)	211 (56.3)	375	
Level of education	Primary/s/t	93 (54.1)	79 (45.9)	172	0.035
	Secondary	158 (44.6)	196 (55.4)	354	
	Tertiary	101 (54.6)	84 (45.4)	185	
Relationship status	Single/m	44 (37.6)	73 (62.)	117	< 0.0001
	Married	211 (62.8)	125 (37.2)	336	
	Divorced	8 (80.0)	2 (20.0)	10	
	Widowed	8 (61.5)	5 (38.5)	13	
	In a relationship	81 (34.5)	154 (65.5)	235	
Number of children alive	None	13 (17.8)	60 (82.2)	73	< 0.0001
	1 or 2	172 (42.2)	236 (57.8)	408	
	More than 2	167 (72.7)	63 (27.3)	230	
Time since last delivery	< 4 weeks	88 (44.3)	111 (55.7)	199	0.058
	4–6 weeks	75 (44.9)	92 (55.1)	167	
	6–10 weeks	94 (54.6)	78 (45.4)	172	
	10–12 weeks	95 (54.9)	78 (45.1)	173	
Knowledge of partner's HIV status	No	89 (35.2)	164 (64.8)	253	< 0.0001
	Yes	263 (57.4)	195 (42.5)	458	
Pregnancy intentions	Within 2 years	5 (27.8)	1372.2 ()	18	< 0.0001
	2–5 years	47 (29.0)	113 (71.0)	160	
	Never again	176 (64.7)	96 (35.3)	272	
	Not sure	124 (47.5)	137 (52.5)	261	
Planned pregnancy	No	185 (49.0)	192 (51.0)	377	0.274
	Yes	162 (53.3)	142 (46.7)	304	
Satisfaction with sterilisation	Dissatisfied/v	1 (33.3)	2 (66.7)	3	0.799
	Satisfied	1 (50.0)	1 (50.0)	2	
	Very satisfied	9 (81.8)	2 (18.2)	11	

### Current use of contraception

**FIGURE 1:**
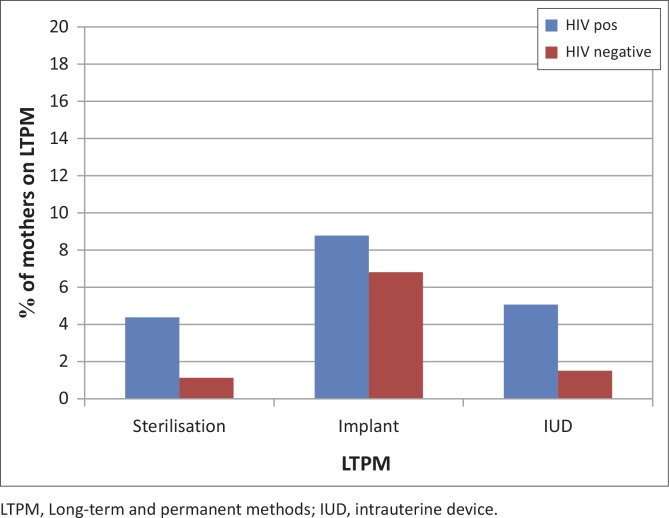
Long-term and permanent methods method use in HIV-positive and HIV-negative post-partum mothers before counselling.

Current contraception use was higher in HIV-positive mothers (84.1%) than in HIV-negative mothers (72.4%) (Table 2). Most of the women (90.5% HIV-negative and 86.2% HIV-positive mothers) were using short-term contraception. The 2-monthly and 3-monthly injectables were the most commonly used methods, with relatively low condom use in both groups. Very few women (7.0% HIV-negative and 15.3% HIV-positive mothers) were on long-term methods, which included IUD, implant and sterilisation (Figure 1), with significantly more HIV-positive mothers on LTPM than HIV-negative mothers before the counselling intervention (Table 3).

**TABLE 2:** Current contraceptive use in HIV-positive and HIV-negative post-partum women.

Characteristic	Value	HIV-positive *n* (%)	HIV-negative *n* (%)	*n*	*p*-value
Total participants	-	352	358	710	-
Current contraceptive use	No	56 (15.9)	99 (27.6)	155	< 0.0001
	Yes	296 (84.0)	259 (72.3)	515	
Current contraception method	Barrier	50 (16.8)	57 (21.5)	107	< 0.0001
	Combined pill	26 (8.7)	24 (9.1)	50	
	Combined injectable	59 (19.9)	59 (22.4)	118	
	Progesterone-only pill	29 (9.8)	38 (14.4)	67	
	Progesterone injectable	78 (26.3)	61 (23.1)	139	
	IUD	15 (5.1)	4 (1.5)	19	
	Implant	26 (8.8)	18 (6.8)	44	
	Sterilisation	13 (4.4)	3 (1.1)	16	
Reasons for using method	Easy to use	142 (47.8)	122 (46.2)	264	< 0.0001
	Efficacy	44 (14.8)	49 (18.6)	93	
	Low risk of side-effects	30 (10.1)	45 (17.1)	75	
	Recommended by health worker	81 (27.2)	48 (18.1)	129	

**TABLE 3:** Prior long-term and permanent methods counselling and preferred method of contraception after counselling intervention.

Variable	Response	HIV-positive (*N* =352) *n* (%)	HIV-negative (*N* =359) *n* (%)	*p-*value
Prior counselling on sterilisation	No	120 (34.3)	196 (57.9)	< 0.0001
	Yes	229 (65.6)	142 (42.0)	
Prior counselling on implant	No	54 (15.5)	93 (27.5)	< 0.0001
	Yes	294 (84.4)	245 (72.6)	
Prior counselling on IUD	No	101 (28.8)	152 (45.01)	< 0.0001
	Yes	249 (71.1)	86 (53.0)	
Preferred method after counselling	Barrier	40 (11.5)	50 (14.0)	< 0.0001
	Combined pill	8 (2.3)	20 (5.6)	
	Combined injectable	58 (16.7)	60 (16.9)	
	Progesterone-only pill	13 (3.8)	15 (4.2)	
	Progesterone injectable	81 (23.3)	64 (18.0)	
	IUD	21 (6.1)	14 (3.9)	
	Implant	67 (19.3)	115 (32.3)	
	Sterilisation	59 (17.0)	18 (5.1)	

Ease of use was an important factor in the choice of contraception for many women (46.2% of HIV-negative and 47.8% of HIV-positive women). More HIV-negative women (17.1%) than HIV-positive women (10.1%) chose contraception methods because of the low risk of side-effects. More HIV-positive women (27.2%) than HIV-negative women (18.2%) chose their method following advice from health workers (Table 2).

Education increased the likelihood that participants would prefer LTPM after counselling (Table 4). Thirty-nine per cent of those with primary schooling, 41% of those with secondary schooling, and 61% of those with tertiary education preferred LTPM after counselling.

**TABLE 4:** Long-term and permanent methods preference after counselling, and level of education of mothers.

Preference	Primary *n* (%)	Secondary *n* (%)	Tertiary *n* (%)
LTPM preference after counselling	65 (39)	145 (41)	112 (64)
**Total**	**168**	**351**	**184**

LTPM, Long-term and permanent methods.

### Counselling on long-term and permanent methods

**FIGURE 2:**
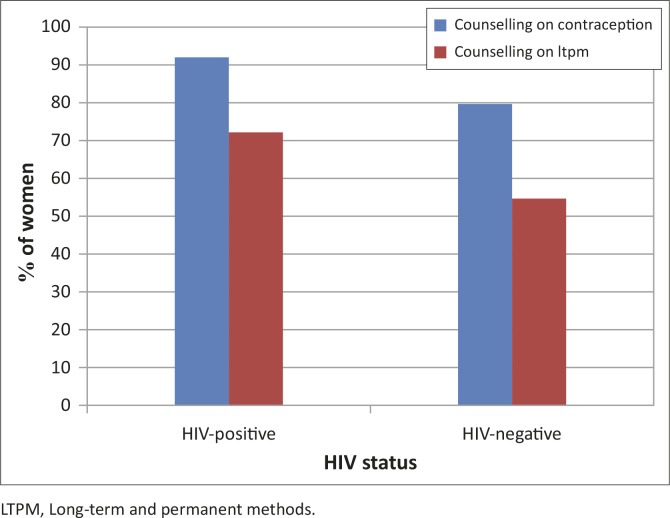
Prior contraceptive and long-term and permanent methods counselling in HIV-positive and HIV-negative post-partum women.

Most of the mothers had received contraceptive counselling during their recent pregnancy, with more counselling received by HIV-positive women (93.7%) than HIV-negative women (81.4%) (Figure 2). HIV-positive women also received more counselling than HIV-negative mothers on IUDs (71.1% and 55.0%) and implants (84.5% and 72.5%, respectively). Although very few women had undergone sterilisation, more HIV-positive (65.6%) than HIV-negative (42.0%) mothers reported prior counselling on sterilisation. Unintended pregnancies were high in both the groups (53.3% in HIV-positive and 57.5% in HIV-negative mothers), with no significant difference between them. Only 13.3% of HIV-positive mothers who had undergone sterilisation regretted it, compared with 25% in HIV-negative mothers. There was no significant difference in the levels of satisfaction between the two groups (Table 1).

Only 1.4% of HIV-positive and 3.6% of HIV-negative mothers wanted to conceive within 2 years, and 50% of HIV-positive mothers and 26.7% of HIV-negative mothers did not want to become pregnant again (Table 1).

### Long-term and permanent methods before counselling

Before counselling, 15.3% of HIV-positive mothers used LTPM compared with 7.0% in HIV-negative mothers, with a statistically significant difference (Table 5).

**TABLE 5:** Long-term and permanent methods use before and preference after counselling.

Methods	HIV-positive *n* (%)	HIV-negative *n* (%)	Total	*p*-value
LTPM use before counselling	54 (15.3)	25 (7.0)	79	0.003
LTPM preference after counselling	147 (41.8)	147 (40.9)	294	0.872

LTPM, Long-term and permanent methods.

### Long-term and permanent methods preference after counselling

**FIGURE 3:**
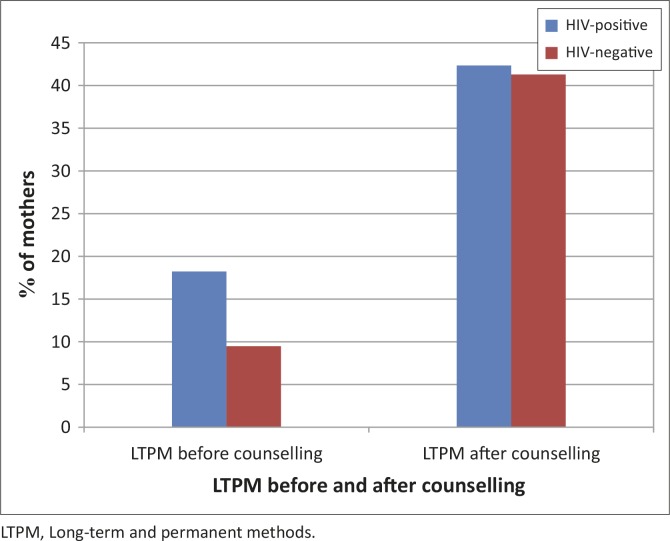
Long-term and permanent methods use before counselling and long-term and permanent methods preference after counselling in HIV-positive and HIV-negative mothers.

After counselling, 42.4% of HIV-positive mothers and 41.3% of HIV-negative mothers preferred to be on LTPM (Figure 3, Tables 3 and 5).

### Factors associated with current use of long-term and permanent methods

In the univariate analysis, marital status, HIV status, counselling on future fertility, age, the number of children alive, time since last pregnancy and pregnancy intentions were all associated with being on LTPM. In the logistic regression, only previous counselling on future fertility and pregnancy intentions were significantly associated with being on LTPM. Previous counselling on fertility had a logistic regression coefficient of 0.91 and a *p*-value of 0.002, indicating that participants who had prior fertility counselling were 2.5 times more likely to be on LTPM than those who did not have prior counselling. Mothers who wanted to wait for more than 2 years or did not want to become pregnant were 1.25 times more likely to be on LTPM than those who wanted to become pregnant within 2 years, with a coefficient of 0.23 and a *p-* value of 0.045 (Tables 6 and 7).

**TABLE 6:** Univariate analysis of factors associated with being on long-term and permanent methods before counselling intervention.

Variable	Odds ratio	*p*-value	Confidence interval
Pregnancy intention	0.61	0.001	0.51–0.75
Time since last pregnancy in weeks	1.29	0.029	1.02–1.62
Number of children alive	2.44	0.001	1.55–3.80
Counselling on future fertility	3.90	0.001	2.29–6.30
Previous use of PMTCT services	2.13	0.003	1.29–3.54
Marital status	0.79	0.007	0.66–0.93
Age	1.08	0.0001	1.05–1.10
Previous contraceptive counselling	1.89	0.23	0.66–5.40
Level of education	1.25	0.179	0.90–1.80

LTPM, Long-term and permanent methods.

**TABLE 7:** Logistic regression of factors associated with use of long-term and permanent methods prior to counselling.

Variable	Regression coefficient	Odds ratio	*p*-value	Confidence interval
Pregnancy intention	−0.2335	0.79	0.045	0.63–0.99
Time since last pregnancy in weeks	0.1832	1.20	0.146	0.93–1.53
Number of children alive	−0.036	1.00	0.991	0.54–1.80
Counselling on future fertility	0.9105	2.49	0.002	1.39–4.46
Previous use of PMTCT	0.2329	1.26	0.412	0.72–1.06
Marital status	0.1338	0.8748	0.181	0.72–1.06
Age in years	0.0446	1.046	0.056	0.99–1.09

LTPM, Long-term and permanent methods.

### Factors associated with long-term and permanent methods preference after counselling

In the univariate analysis, participants’ age, future pregnancy intentions, prior LTPM use, level of education and the number of children alive were all associated with LTPM preference after counselling. In the logistic regression, only the level of education and prior LTPM use were associated with LTPM preference after counselling. Those who were on LTPM were 12 times more likely to prefer it after counselling than those who were not (Tables 8 and 9).

**TABLE 8:** Univariate analysis of factors associated with long-term and permanent methods preference after counselling.

Variable	Odds ratio	*p*-value	Confidence interval
Age in years	1.04	0.001	1.03–1.06
Level of education	1.59	0.0001	1.30–1.98
Employment status	0.99	0.862	0.83–1.16
Marital status	1.00	0.864	0.91–1.10
Partner's status	0.87	0.38	0.63–1.19
LTPM use before counselling	13.27	0.001	5.9–29.5
Pregnancy intention	0.80	0.001	0.71–0.81
Number of children alive	1.34	0.017	1.05–1.71

LTPM, Long-term and permanent methods.

**TABLE 9:** Logistic regression of factors associated with use of long-term and permanent methods after counselling.

Variable	Odds ratio	*p*-value	Confidence interval
Age in years	1.00	0.716	0.97–1.04
Level of education	1.48	0.04	1.13–1.97
Number of children alive	0.77	0.227	0.51–1.21
LTPM use before counselling	12.3	0.0001	5.45–27.3
Pregnancy intention	0.870	0.085	0.75–1.90

LTPM, Long-term and permanent methods.

## Discussion

LTPM use was very low in both HIV-positive and HIV-negative mothers, which is consistent with prior studies in Zambia and South Africa.^1,15^ As in the other studies, our results show that a high percentage of women were on injectable contraceptives, citing ease of use and convenience as important reasons for choosing their methods. We found, however, that LTPM use before counselling was significantly higher in HIV-positive mothers, which was not reported in the earlier studies. This finding is not unexpected in our setting, where most health workers still believe that HIV-positive women should not become pregnant because of their sero-status and recommend long-term or permanent methods. Counselling on all LTPMs was significantly higher in HIV-positive than HIV-negative mothers. More than 65% of women had counselling on an LTPM method prior to the study, but only 14.1% used LTPM. After the trained interviewer's counselling on all available contraceptives, there was a significant increase in the number of women preferring to be on LTPM. In the South African study, 78% of mothers preferred to be on LTPM after the counselling intervention, which demonstrates that mothers are willing to be on LTPM if it supports their fertility desires.

LTPM use before counselling was associated with prior counselling on future fertility and pregnancy intentions, which was in agreement with the South African study.^15^ Post-counselling LTPM preference was associated with the level of education and the current use of LTPM. The more educated the women, the more they preferred to be on LTPM, which was also in agreement with the above-cited South African study.

However, even after counselling, only 6.1% of HIV-positive women and 3.9% of HIV-negative women preferred to use an IUD. Previous studies^2^ have cited fear of infection and procedures involved^15^ as the main reasons why women, especially those who were HIV-positive, shunned IUDs. These fears were addressed in the counselling sessions. The IUD has been found to be safe in HIV-positive mothers who are healthy,^23^ is very convenient, with a quick return to fertility after removal^24^ and does not require repeated contact with health services. It is potentially more reliable than injectables, which women may forget to return for, or suspend the use of.^25^

The high unintended pregnancy rates in HIV-positive and HIV-negative mothers suggest that there is still an unmet need. Despite the high levels of counselling on contraception, LTPM counselling levels were lower, particularly in HIV-negative women. LTPM appears to be a lower priority in counselling sessions by health workers, which could contribute to the poor uptake of this method.

Those who wanted to delay pregnancy by more than 2 years or did not want another child were more likely to be on LTPM. Prior counselling on future fertility was also associated with being on LTPM. This finding was in agreement with the South African study,^10^ which showed higher chances of being on LTPM for mothers who had prior counselling on future fertility. Consequently, there is a need for broader counselling in post-partum mothers to include their future fertility intentions and all contraceptives available to reduce unintended pregnancy rates.

### Limitations

Because of fear of reprimand by nurses who acted as data collectors, some of the mothers might have given false responses, especially on future pregnancy intentions and on the current use of contraceptives. The expressed preferred contraceptive method of participants after counselling was one of our main outcomes, but this may, however, not translate into actual use by the participants.

Because we used a convenience sample, there could be other confounding factors leading to spurious results. Contraceptive use could be different in clients who present during a different time period than the one used for data collection. In addition, our interviewers were not blinded and hence could have biased the study results. Staff nurses who acted as data collectors could also have introduced bias because they were working at the same hospital. They could easily interact with nurses working in these departments, causing them to change the way they offer contraceptive counselling and contraceptives.

As a before-and-after study without a control, there might have been other factors that influenced the mothers outside of our counselling intervention, which we did not measure. If we had had a control group, we might have detected that the routine consultations could in fact also have had an impact on LTPM uptake.

Our follow-up period was short, and hence we were unable to confirm whether the changes in contraceptive preference and uptake of LTPM were sustained and whether this translated into a reduction in unplanned pregnancies.

### Implications for future practice and research

Future efforts should focus on increasing women's knowledge on safe, long-term contraceptive methods so that they can make informed choices. Future research should use control groups to assess whether these interventions really increase LTPM and reduce unintended pregnancy rates.

## Conclusion

Although the reported use of contraceptives was very high, LTPM uptake was still very low, which was reflected in unintended pregnancies that were very high in both HIV-positive and HIV-negative mothers, suggesting that strategies to prevent unwanted pregnancies and vertical transmission of HIV in HIV-positive mothers need strengthening. Women who were counselled on, and offered a wider range of, contraception services expressed a high preference for future use of LTPM. Prior to the intervention, women who had been previously counselled on future fertility intentions were more likely to use LTPM than those who had not been counselled. This finding shows the importance of counselling mothers on the uptake of LTPM. Post-counselling, current use of LTPM and level of education were associated with a preference for LTPM, indicating that promoting women's education can also go a long way to reduce unmet needs in contraception, and hence unintended pregnancies. Most of the women who were on LTPM still preferred LTPM post-counselling, showing that this method is convenient and suited most mothers’ fertility intentions, who wanted to wait for more than 2 years before becoming pregnant.

Of concern were the low IUD preference levels even after counselling. If promoted actively, this method would be very important in this setting where most mothers are willing to wait for more than 2 years without becoming pregnant. Further research on the acceptability of interventions to increase IUD uptake is needed in high HIV-prevalence settings.

We recommend that a study with a longer follow-up be conducted to assess whether the effects of the intervention were sustained and whether increased use of LTPM would reduce unintended pregnancies.
